# Sonographic Presentation of the Geyser Sign

**DOI:** 10.1155/2019/5623530

**Published:** 2019-10-31

**Authors:** Syed Amir Gilani, Riffat Mehboob, Raham Bacha, Aima Gilani, Iqra Manzoor

**Affiliations:** ^1^Faculty of Allied Health Sciences, The University of Lahore, Lahore, Pakistan; ^2^Combined Military Hospital, Lahore, Pakistan

## Abstract

A case of geyser sign with acromioclavicular (AC) joint cyst with underlying rotator cuff tear is presented. Ultrasound modality is used to diagnose the clinical case of the AC cyst with positive geyser sign. A 91-year-old male patient presented with a complaint of right-sided shoulder and neck pain. The physical examination revealed a large lump on his right shoulder with restricted shoulder movements. During ultrasound examination, a chronic supraspinatus tendon (ST) tear and AC joint cyst with a positive geyser sign was observed. The ultrasound diagnosis was also confirmed with magnetic resonance imaging (MRI). AC joint cyst with geyser sign is a rare condition. Few cases have been published with this type of pathology. To our knowledge, this is the first report of its kind from Pakistani population.

## 1. Introduction

Acromioclavicular (AC) joint cyst is a rare abnormality that results from the chronic rotator cuff tear. The cyst is composed of a thick viscous fluid and is enveloped by a fibrous capsule. During arthrography, the fluid is erupting from the superior aspect of the AC joint which is termed as the “geyser sign” [1]. The pathophysiology of the cyst is related with the rotator cuff tear and the increased amount of fluid of the tear. This fluid escapes through the defect in the AC joint capsule which acts as a one-directional valve. The cysts are mostly painless and lies over the AC joint [1].

Shoulder impingement and rotator cuff tear are the main causes of the disability of the shoulder and immobilization of the arm. They can be misdiagnosed easily, and if not diagnosed properly, it may lead to mismanagement and aggravation of the disease [2]. The signs and symptoms of the supraspinatous tendon (ST) tear are highly variable ranging from the asymptomatic to restricted shoulder movements due to pain, even the patient is sometimes not able to move the arm due to severe pain [3]. The symptoms can also be associated with the degree of tear, age of the patient, and several other abnormalities related with it, for example, rheumatoid arthritis, septic arthritis, and osteoarthritis [4]. The drop arm test and Jobe's test are thought to be helpful in the diagnosis of the ST tear. Acute and chronic types of rotator cuff tear can easily be differentiated with the help of musculoskeletal ultrasound. The acute rotator cuff tear is mostly seen in the young population and usually do not have bony degeneration (bony spurs); defects in the tendon fibers usually appear hypoechoic to anechoic on ultrasound examination, acute rotator cuff tear most commonly occurs in the anterior aspect of the ST, while the chronic rotator cuff tear occurs in the posterior part of the ST, with dispersed calcification and sometimes posterior acoustic shadowing [5].

Different modalities have been used for the diagnosis of the AC joint cysts and rotator cuff tear with geyser sign including computed tomography (CT) scan, MRI, arthrography, ultrasound, and so on. MRI is the first line of modality in the diagnosis of the AC joint cysts and rotator cuff tear with geyser sign [6]. But in this case report, the cyst of the AC joint with geyser sign is diagnosed with the help of musculoskeletal ultrasound.

## 2. Case Presentation

A 91-year-old male visited our clinic with right-sided shoulder and neck pain. He was a coach of cricket team by profession. On physical examination, a lump on his right shoulder was found just above the AC joint. He had the history of lump since one year but did not feel any pain, and it continued to enlarge. The lump was nontender, nonmobile, firm, and painless. He felt difficulty in raising his right arm and restricted shoulder movements. Even he was not able to lie on his right side due to pain. There was no history of fever, weight loss, or other symptoms. The medical history was unremarkable, and there was no history of previous surgery on the affected shoulder.

**Figure 1:**
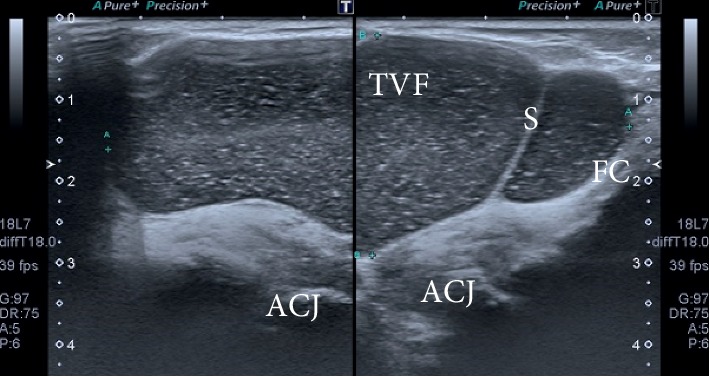
Supraspinatus tear with geyser sign. TVF, thick viscous fluid; S, septa; FC, fibrous capsule; ACJ, acromioclavicular joint.

**Figure 2:**
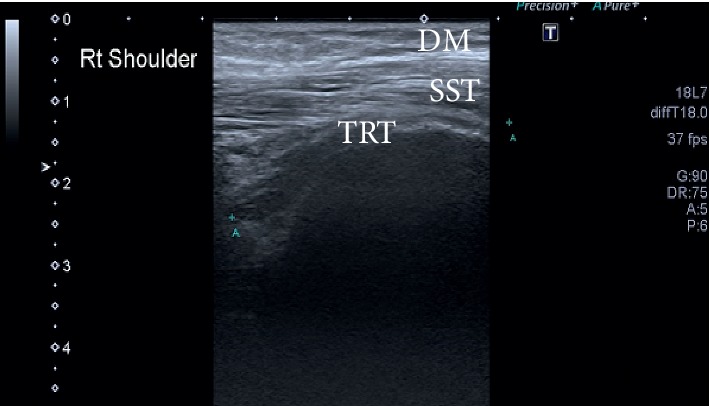
Supraspinatus tendon tear with retraction. DM, deltoid muscle; SST, supraspinatous tendon; TRT, tear with retraction of tendon.

**Figure 3:**
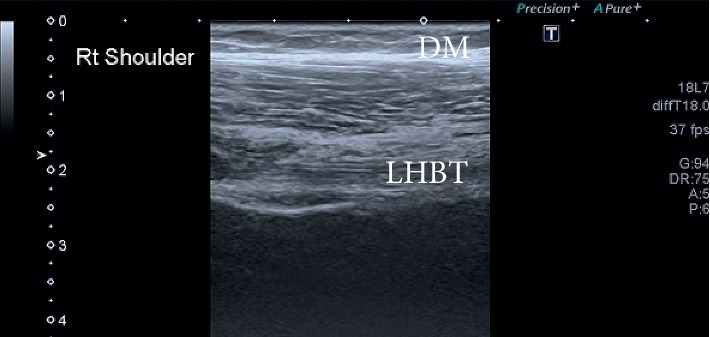
Long head of biceps tendon. DM, deltoid muscle; LHBT, long head of bicep tendon.

During ultrasound examination, the lump was revealed as cystic lesion just above the AC joint. The size of the cyst was 8.0 × 4.5 × 2.1 cm with posterior acoustic enhancement. The cyst was anechoic with acoustic streaming and has communication with the ST (Figure 1). Furthermore, the attenuation was seen in the subscapularis tendon. The posterior aspect of the ST was seen hypoechoic with dispersed calcification and posterior acoustic shadowing, suggestive of full-thickness chronic ST tear (Figure 2). The tear was associated with bony spurs (degenerative changes). A small effusion was seen in the glenohumeral (GH) space, having communication with the subacromial bursa. On real time ultrasound examination, a synovial fluid was observed escaping from the rotator cuff defect across the subacromial bursa and decompressing superiorly through the AC joint. This type of fluid eruption is termed as “geyser sign” by the radiologist. The long head of biceps tendon was present within the bicipital groove and appeared hypoechoic suggesting an element of tendinopathy (Figure 3).

**Figure 4:**
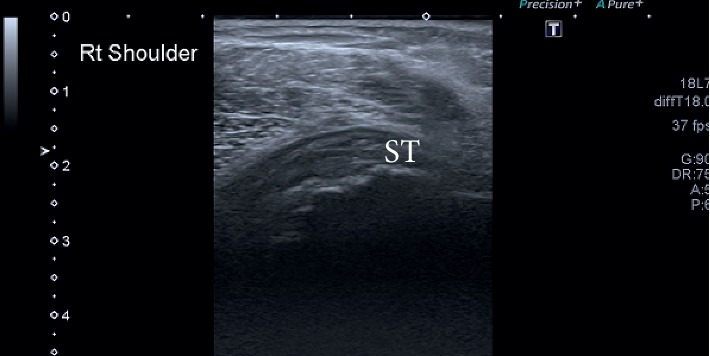
Subscapularis. ST, subscapularis tendon.

**Figure 5:**
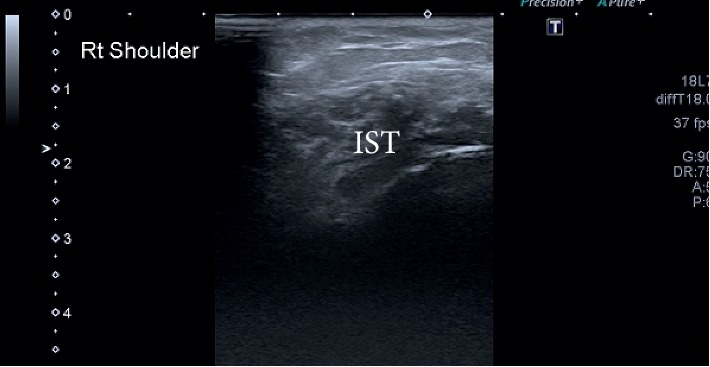
Infraspinatus. IST, infraspinatus tendon.

**Figure 6:**
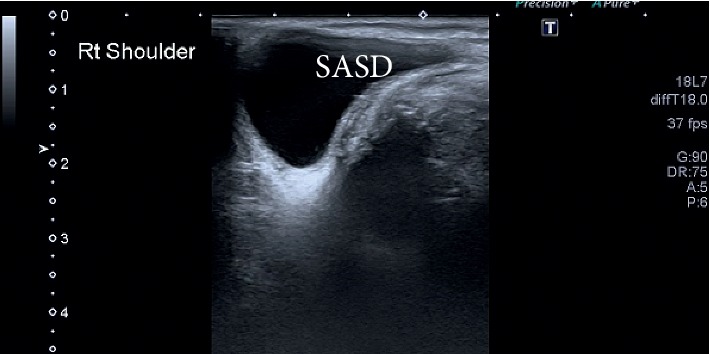
Subacromial-subdeltoid bursitis, thick walls with internal echoes. SASD, subacromial-subdeltoid.

The subscapularis and infraspinatus tendon demonstrated decreased echogenicity and loss of the usual fibrillar echotexture suggesting tendinopathy (Figure 4). The infraspinatus tendon also appeared irregular, suggesting detachment of a few fibers (Figure 5). There was a complete tear (full thickness and full width) of the ST with retraction of 4 cm. Marked distension of the subacromial-subdeltoid bursa was observed (Figure 6), and the humeral head was also irregular. The swelling over the apex of the shoulder corresponded to a large cystic collection with internal echoes. The fluid was continuous with the AC joint. This presentation is commonly associated with a complete tear of the ST.

MRI was then performed for the confirmation of the ultrasound diagnosis. The mass was visible as hyperintense on T2-weighted scan and hypointense on T1-weighted scan. The fluid was observed in the GH space having communication with the right subacromial bursa. A full-thickness tear in the posterior part of ST was also observed. The lesion appeared as cystic with peripheral enhancement.

## 3. Discussion

Acromioclavicular joint cyst is a rare clinical finding, which is found secondary after the presence of chronic rotator cuff tear. The cyst is formed due to the escape of fluid from the tear into the AC joint [7]. AC joint cyst is mostly painless, and only a firm mass is palpable just above the AC joint. Aspiration of the cyst is not preferred because it has the potential of reoccurrence and has the risk of the formation of fistula due to aspiration [8]. Very few research studies have been found in the literature related to AC joint cyst and geyser sign. Up till 2010, only 41 cases have been published related to the AC joint cyst. In 5 cases, the cyst was found with intact musculature of the rotator cuff. In remaining of the 36 cases, the cyst was found with complete rotator cuff tear. According to this literature review, the cyst was found most commonly in males (31 cases) as compared with females (10 cases). The mean age in cases was 69.47 with a range of 51–90 years. Only 3 studies had diagnosed the cases on the basis of ultrasound for the AC joint cyst, while the rest of the studies used MRI and arthrography as a diagnostic modality [9].

Craig EV was the first researcher to discover the AC joint cyst with geyser sign during the arthrogram. He published 2 articles related to this pathology, one in 1984 and the second in 1986 [10, 11]. Two types of the AC joint cysts are described in a review article by Hiller et al. in 2010. In type 1, there is no communication with the GH joint while in type 2 due to rotator cuff tear, there is a communication established between the AC and GH joints. Both these types of cysts can be managed operatively and nonoperatively, depending upon the age, medical condition, level of tear, and symptoms of the patient [9]. Montet et al. described the AC joint cyst located within the trapezius muscle which was diagnosed with the help of ultrasound and MRI [12].

Previous case reports also confirmed that AC joint cyst is mostly found in males and in elderly population. Ultrasound modality was used for the diagnosis of the cyst in the current study, and confirmation was done with the help of MRI due to its high sensitivity, specificity, and accuracy.

## 4. Conclusion

Acromioclavicular joint cyst is a rare clinical disorder. Geyser sign provides a very valuable information about the integrity of the supraspinatus tendon tear. This sign usually occurs due to large, full-thickness and chronic rotator cuff tear. The demonstration of the geyser sign during the assessment of a shoulder lump for presence of malignancy indicates a benign process.

## References

[1] Zhang Y., Old J. (2018). Massive acromioclavicular joint cyst with intramuscular extension: case report and review. *Case Reports in Orthopedics*.

[2] Fukuda H., Hamada K., Nakajima T., Yamada N., Tomonaga A., Goto M. (1996). Partial-thickness tears of the rotator cuff. *International Orthopaedics*.

[3] Mohammad S., Raham Bacha D., Ullah H., Manzoor I. (2018). Sonographic comparison of patellar tendon thickness with height in chronic diabetic patients and normal individuals. *Research & Reviews: Orthopedics journal*.

[4] Laver L., Lubiatowski P., Zumstein M. A., Landreau P. (2018). Shoulder instability in handball. *Handball Sports Medicine: Basic Science, Injury Management and Return to Sport*.

[5] Manzoor I., Bacha R., Gilani S. A., Liaqat M. (2019). The role of ultrasound in shoulder impingement syndrome and rotator cuff tear. *Annals of Orthopaedics, Trauma and Rehabilitation*.

[6] Cooper H. J., Milillo R., Klein D. A., DiFelice G. S. (2011). The MRI geyser sign: acromioclavicular joint cysts in the setting of a chronic rotator cuff tear. *American Journal of Orthopedics*.

[7] Gumina S., Candela V., Passaretti D. (2016). Acromioclavicular joint cyst in ASA 3-4 patients. Whether and how quickly it recurs after aspiration and steroid injection. *Acta Orthopaedica Belgica*.

[8] Murena L., D’angelo F., Falvo D. A., Vulcano E. (2009). Surgical treatment of an aseptic fistulized acromioclavicular joint cyst: a case report and review of the literature. *Cases Journal*.

[9] Hiller A. D., Miller J. D., Zeller J. L. (2010). Acromioclavicular joint cyst formation. *Clinical Anatomy*.

[10] Craig E. V. (1984). The geyser sign and torn rotator cuff: clinical significance and pathomechanics. *Clinical Orthopaedics and Related Research*.

[11] Craig E. V. (1986). The acromioclavicular joint cyst: an unusual presentation of a rotator cuff tear. *Clinical Orthopaedics and Related Research*.

[12] Montet X., Zamorani-Bianchi M. P., Mehdizade A., Martinoli C., Bianchi S. (2004). Intramuscular ganglion arising from the acromioclavicular joint. *Clinical Imaging*.

